# Generation and analysis of mouse embryonic stem cells with knockout of the Mcph1 (microcephalin) gene

**DOI:** 10.18699/vjgb-24-55

**Published:** 2024-09

**Authors:** A.M . Yunusova, A.V. Smirnov, T.A. Shnaider, I.E. Pristyazhnuk, S.Y. Korableva, N.R. Battulin

**Affiliations:** Institute of Cytology and Genetics of the Siberian Branch of the Russian Academy of Sciences, Novosibirsk, Russia; Institute of Cytology and Genetics of the Siberian Branch of the Russian Academy of Sciences, Novosibirsk, Russia; Institute of Cytology and Genetics of the Siberian Branch of the Russian Academy of Sciences, Novosibirsk, Russia; Institute of Cytology and Genetics of the Siberian Branch of the Russian Academy of Sciences, Novosibirsk, Russia; Novosibirsk State University, Novosibirsk, Russia; Institute of Cytology and Genetics of the Siberian Branch of the Russian Academy of Sciences, Novosibirsk, Russia Novosibirsk State University, Novosibirsk, Russia

**Keywords:** Mcph1/microcephalin, chromosome condensation, mESCs, gene expression analysis, Mcph1 (микроцефалин), конденсация хромосом, ЭС клетки мыши, транскриптомный анализ

## Abstract

Chromatin is not randomly distributed within the nucleus, but organized in a three-dimensional structure that plays a critical role in genome functions. Сohesin and condensins are conserved multi-subunit protein complexes that participate in mammalian genome organization by extruding chromatin loops. The fine temporal regulation of these complexes is facilitated by a number of other proteins, one of which is microcephalin (Mcph1). Mcph1 prevents condensin II from associating with chromatin through interphase. Loss of Mcph1 induces chromosome hypercondensation; it is not clear to what extent this reorganization affects gene expression. In this study, we generated several mouse embryonic stem cell (mESC) lines with knockout of the Mcph1 gene and analyzed their gene expression profile. Gene Ontology analyses of differentially expressed genes (DEGs) after Mcph1 knockout revealed gene categories related to general metabolism and olfactory receptor function but not to cell cycle control previously described for Mcph1. We did not find a correlation between the DEGs and their frequency of lamina association. Thus, this evidence questions the hypothesis that Mcph1 knockout-mediated chromatin reorganization governs gene expression in mESCs. Among the negative effects of Mcph1 knockout, we observed numerous chromosomal aberrations, including micronucleus formation and chromosome fusion. This confirms the role of Mcph1 in maintaining genome integrity described previously. In our opinion, dysfunction of Mcph1 may be a kind of “Rosetta stone” for deciphering the function of condensin II in the interphase nucleus. Thus, the cell lines with knocked-out Mcph1 can be used to further study the influence of chromatin structural proteins on gene expression.

## Abstract (ru)

Хроматин в ядре клетки распределен не хаотично, а имеет организованную структуру, которая оказывает прямое влияние на функционирование генома. Одними из основных архитектурных белков хроматина в клетках млекопитающих являются консервативные мультисубъединичные белковые комплексы: когезин и конденсины. Эти комплексы способны протягивать петли хроматина, опосредуя контакты между удаленными участками ДНК. Тонкая временная регуляция их активности осуществляется рядом других белков, один из которых – микроцефалин (Mcph1). Mcph1 препятствует взаимодействию конденсина II с хроматином в интерфазе. При нарушении его функции наблюдается масштабная реорганизация хроматина, вызванная аномальной загрузкой конденсина II. Как это сказывается на экспрессии генов, до сих пор неизвестно. В данном исследовании мы создали несколько линий эмбриональных стволовых клеток мыши с нокаутом гена Mcph1, охарактеризовали их и проанализировали профиль экспрессии генов. Аннотация дифференциально экспрессирующихся генов в терминах генной онтологии выявила категории генов, относящиеся к общему метаболизму и функционированию обонятельных рецепторов, но не к регуляции клеточного цикла, описанной ранее для Mcph1. Мы также не обнаружили корреляции между генами, изменившими свою транскрипционную активность после нокаута Mcph1, и вероятностью их локализации на ядерной ламине. Этот результат ставит под сомнение гипотезу о влиянии опосредованной нокаутом Mcph1 архитектуры хроматина на экспрессию генов. Среди негативных эффектов нокаута Mcph1 мы наблюдали множественные хромосомные аберрации, включая нарушения сегрегации хромосом с образованием микроядер, а также слияние хромосом. Это подтверждает описанную в предыдущих исследованиях роль белка Mcph1 в поддержании целостности структуры генома. Мы полагаем, что нокаут Mcph1 может оказаться своеобразным «розеттским камнем», способным расшифровать функции конденсина II в интерфазном ядре. Полученные нами линии эмбриональных стволовых клеток с нокаутом гена Mcph1 могут быть использованы для дальнейшего изучения влияния структурных белков хроматина на экспрессию генов.

## Introduction

**Fig. 1.**
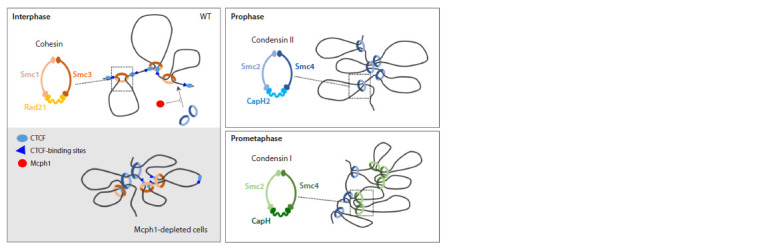
DNA loop extrusion by SMC (Structural maintenance of chromosomes) complexes during cell-cycle progression in WT and Mcph1-depleted cells.

The three-dimensional organization of chromatin plays a crucial role in maintaining genome stability and regulating key cellular processes such as DNA replication, DNA repair, and gene expression (Marchal et al., 2019; Stadhouders et al., 2019; Sanders et al., 2020). Interphase chromosomes are decondensed and distributed all over the nucleus. Contacts between distant genomic regions are important in the regulation of gene expression and mediated by CTCF and cohesin complexes (SMC family of ATPases) (Dixon et al., 2012; Rao et al., 2014) (Fig. 1). The transition from interphase to mitosis leads to significant chromatin structure changes: chromosomes become highly compacted due to the loading of condensin complexes – other members of the SMC protein family (Earnshaw, Laemmli, 1983; Naumova et al., 2013). Condensin II builds large regular chromatin loops early in mitosis forming helically arranged axial scaffold, whereas condensin I generates smaller nested loops inside the large loop and promotes the widening of the chromosomes. As mitosis progresses, outer loops grow and the number of loops per turn increases, promoting axial shortening of the chromosomes (Gibcus et al., 2018) (Fig. 1).

In recent years, interest in condensin complexes as motor proteins involved in establishing chromatin loops has greatly increased driven by advances in 3D genomics and super-resolution microscopy methods. However, many of their functions remain unclear. One of the most intriguing questions is the role of condensin II in the interphase nucleus (Wallace, Bosco, 2013). Unlike cytoplasmic condensin I, which interacts with chromatin only after nuclear envelope breakdown, condensin II is present in the nucleus throughout interphase (Hirota et al., 2004; Ono et al., 2004). Some studies suggest that condensin II loads coordinately with cohesin and transcription factor TFIIIC onto chromatin at the promoters of actively transcribed genes (Dowen et al., 2013; Yuen et al., 2017). Other studies indicated that condensin II does not play any significant role during interphase since the depletion of condensin II in non-dividing cells does not lead to changes in the spatial organization of the genome or gene transcriptional activity (Abdennur et al., 2018; Hoencamp et al., 2021). It is well established that condensin II’s activity during interphase is blocked by microcephalin (Mcph1) (Trimborn et al., 2006; Yamashita et al., 2011; Houlard et al., 2021). Mcph1 is a multifunctional protein that also participates in DNA repair, cell cycle control, apoptosis, and chromatin remodeling (reviewed by (Kristofova et al., 2022)). Mcph1 binds to condensin II through its short linear motif in the central domain thereby blocking the condensin II interaction with chromatin (Houlard et al., 2021). Disruption of Mcph1 function leads to chromosome condensation of interphase nuclei (Fig. 1). As a result, mutant cells acquire a unique phenotype characterized by prophase-like compacted chromosomes during interphase (Neitzel et al., 2002; Gruber et al., 2011).

It has been shown that in mouse embryonic stem cells (mESCs) Mcph1 knockout leads to altered chromatin architecture by enhancing the mixing of A and B chromatin compartments. This is consistent with microscopic observations – highly condensed chromosomes become “individualized” in the interphase nuclei, while the chromocenters have disappeared (Houlard et al., 2021). Whether these chromatin state changes can affect gene expression is not clearly understood. To address this issue, we generated mESCs with stable Mcph1 knockout and analyzed the changes in gene expression profiles

## Materials and methods

Mouse embryonic stem cells culture. All ΔMcph1 cell lines were generated from mouse ES cells (Rad21-miniIAA7- eGFP) previously established in our laboratory (Menzorov et al., 2019; Yunusova et al., 2021). Cells were cultured on the plates coated with a 1 % gelatin solution under 2i conditions, which ensures the pluripotency by specifically blocking the MAPK–ERK pathway (PD0325901, 1 μM) and glycogen synthase kinase 3 (CHIR99021, 3 μM) in DMEM (Thermo Fisher), supplemented with 7.5 % ES FBS (Gibco), 7.5 % KSR (Gibco), 1 mM L-glutamine (Sigma), NEAA (Gibco), 0.1 mM β‐mercaptoethanol, LIF (1,000 U/ml, Polygen), and 1× penicillin/streptomycin (Capricorn Scientific). The growth medium was changed to a fresh one every day. Upon reaching appropriate confluence (70–80 %), the cells were passaged every 2–3 days.

Gene targeting of the Mcph1 gene in mESCs. The sequences of guide RNAs were taken from the article (Houlard et al., 2021). gRNAs were cloned into the gRNA_cloning vector (Addgene, 41824). For exogenous Cas9 expression, the vector pCSDest2-2XNLS-SpCas9-WT-NLS-3XHA-NLS-TALentry (Addgene, 69232) was used. The plasmids were introduced into cells via electroporation (Neon Transfection System, Thermo Fisher Scientific, USA) as follows: for each 10 μl electroporation, 250,000 cells and 1 μg of total DNA (with an equimolar ratio of the two vectors) were used. Electroporation was performed following the manufacturer’s protocol under conditions 6 and 10, previously determined as the most optimal for efficiency/survival ratio for mouse ESCs in our laboratory. After electroporation, cells were seeded into a 24-well plate in pre-warmed media without antibiotics. The next day cells were split into 10 cm dishes at low density. The medium was changed every 2–3 days. After single-cell clones were visible, a subset of clones was handpicked with pipette tips under the light microscope and transferred into a drop of trypsin/EDTA in each well of a 96-well plate, and then resuspended in growth medium. Upon reaching a confluent density, subclones were plated into two new 96-well plates (one for stock storage and one for PCR-genotyping).

For PCR-genotyping, cells were lysed in PBND buffer (10 mM Tris-HCl, 50 mM KCl, 2.5 mM MgCl2, 0.45 % NP- 40, and 0.45 % Tween 20, pH 8.3) containing 1 μg/μl proteinase K (NEB, USA) for an hour at 55 °C. After inactivation of proteinase K (95 °C, 10 minutes), 1 μl of lysate was used as a template for PCR amplification of the Cas9-target site. The target region included exon 2 of the Mcph1 gene and was amplified using HS-Taq DNA Polymerase kit (Biolabmix, Russia) under the following conditions: 95 °C for 30 s, followed by 34 cycles of 95 °C – 10 s, 60 °C – 20 s, 72 °C – 1 min, and a final elongation at 72 °C for 5 minutes. The primers used were: Mcph1-del-F – ACCACATGCTTTGGCGTAGA and Mcph1-del-R – GCCAGACTCAAGTCTCCCAC. Amplified DNA fragments were separated on 2 % agarose gel. For selected subclones, amplicons were purified and their nucleotide sequence was determined by Sanger sequencing.

Protein detection by Western Blotting. Growth medium was discarded and cells were washed with PBS and scraped from the surface in the presence of RIPA buffer (50 mM Tris-HCl pH 8, 150 mM NaCl, 1 % Triton X-100, 0.5 % sodium deoxycholate, and 0.1 % SDS) containing the protease inhibitor cocktail [1x Complete ULTRA, 1x PhosSTOP (both from Roche, Switzerland), 5 mM NaF (Sigma-Aldrich, USA)]. After that, the cell lysates were sonicated by three 10 s pulses at 33–35 % power settings with UW 2070 (Bandelin electronics, Germany). The sonicated samples were centrifuged at 14,000 g for 20 min at 2 °C, frozen, and stored at –80 °C. The protein concentrations were quantified according to instruction’s protocol by using Pierce BCA Protein Assay Kit (Thermo Fisher Scientific, USA). Equal amounts of the denatured total protein (20 μg) were separated on 10 % SDSPAGE gel and then transferred onto the Immun-Blot PVDF membrane (Bio-Rad). After blocking in 5 % milk in TBST (50 mM Tris base, 150 mM NaCl, 0.05 % (v/v) Tween 20) for 2 h, the membranes were incubated with primary antibodies against Mcph1 protein (D38G5) Rabbit mAb #4120 (Cell Signaling Technology, USA) at a 1:1,000 dilution overnight at 4 °C. On the following day, after three washes with TBST buffer (10 min) membranes were incubated with horseradish peroxidase – conjugated secondary antibodies (Anti-rabbit IgG #7074, Cell Signaling Technology) for 2 h at room temperature. Detection was performed with Clarity™ (BioRAD, USA) and iBright™ FL1500 (Thermo Fisher Scientific, USA).

RNA extraction and transcriptome sequencing. The isolation of total RNA was performed using Trizol reagent (Sigma-Aldrich, MA, USA) following the manufacturer’s instructions. The isolated RNA samples were resuspended in DEPC-treated water, then RNA concentration and quality were assessed by spectrophotometry and gel electrophoresis. Total RNA was sequenced on the BGISEQ-500 High-throughput Sequencing Platform (BGI, Beijing, China). The expression of RNA transcripts was quantified using Salmon (Patro et al., 2017). All analyses were performed using R Statistical Software (v4.3.2; R Core Team 2023). Raw counts were processed and normalized by Log2 fold change using tximport (https:// github.com/thelovelab/tximport), genefilter (https://github. com/Bioconductor/genefilter), GenomicFeatures (https:// github.com/Bioconductor/GenomicFeatures) and DESeq2 (https://github.com/thelovelab/DESeq2). Volcano plots were constructed using the EnhancedVolcano R package (https://github.com/kevinblighe/EnhancedVolcano). The heat map was generated using the ComplexHeatmap (https://github. com/jokergoo/ComplexHeatmap). The gene ontology term enrichment analysis was carried out using the PANTHER server (https://www.pantherdb.org). The set of genes specifically expressed in mESCs with a base mean level ≥100 was used as a reference set.

To examine whether the differences in gene expression in ΔMcph1 cell lines were associated with the differences in lamina association, we used the LaminB1-DamID libraries from (Borsos et al., 2019). Next, we determined the DamID score in a 100 kb bin containing the coordinates of the transcription start sites of DEGs (Supplementary Material 1)1. To determine the correlation between the DamID score and the magnitude of the change in activity (log2FoldChange), the Pearson correlation coefficient was calculated for each gene.


Supplementary Materials are available in the online version of the paper: https://vavilovj-icg.ru/download/pict-2024-28/appx18.xlsx


Сell cycle analysis by flow cytometry. After trypsinization, cell pellets were washed with PBS and resuspended in cold 70 % ethanol for fixation overnight at 4 °C. The next day, the fixed cells were centrifuged and the fixative was thoroughly removed. The cell pellet was suspended in PI solution (1 % Triton X-100, 500 μg/ml propidium iodide, and 10 μg/ml RNase A in PBS) and incubated for 30 min at room temperature. After that, cell cycle distribution was analyzed using a BD FACS Aria flow cytometer (BD Biosciences, USA).

Chromosome spread analysis. Chromosome preparations were obtained following standard protocols (Matveeva et al., 2017). Briefly, cells were exposed to a 0.1 μg/ml colcemid (Merck, Germany) in growth medium for 3 h. After, cells were treated with 0.05 % Trypsin-EDTA solution (Capricorn Scientific GmbH, Germany), hypotonic solution (0.25 % KCl and 0.2 % sodium citrate) was added directly to the culture plates for 20 min at 37 °C. Then, cells were harvested, fixed with Carnoy fixative (3:1 methanol:glacial acetic acid) and dropped onto cold wet glass slides. Nuclear DNA was counterstained with 1 μg/ml 4′,6-diamidino-2-phenylindole (DAPI) (Sigma- Aldrich, USA). Representative images were captured under a Carl Zeiss Axioscop 2 fluorescence microscope equipped with a CoolCube1 CCD-camera (Meta Systems, Altlussheim, Germany) at the Public Center for Microscopy SB RAS, Novosibirsk. The fraction of PLCs was determined after counting 130 to 230 nuclei in two replicas for each sample. The total length of all chromosomes in 15 metaphases for each sample was measured in arbitrary units (au) using ImageJ software.

Statistical analysis. Data analysis was performed using GraphPad Prism software package, employing two-sided Student’s t test or Analysis of variance (ANOVA). Differences were considered statistically significant at p < 0.05.

## Results

Generation and chromosome analysis of ΔMcph1 cells

**Fig. 2.**
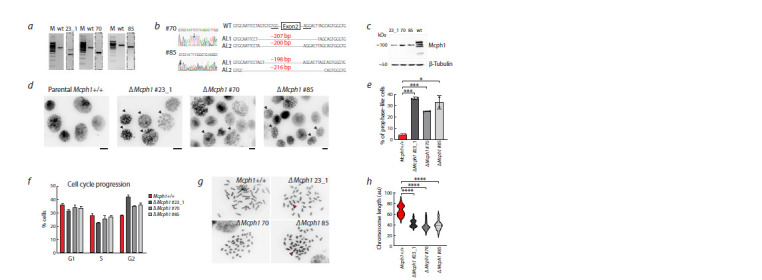
The deletion of Mcph1 in mESCs induces chromosome condensation and metaphase chromosome shortening a, Representative PCR genotyping of genome-edited mESCs clones (three potential clones are shown as an example). b, Genotyping of the potential clones by Sanger sequencing. c, Western blot analysis of parental Mcph1+/+ and ΔMcph1 cell lines. d, Representative images of prophase-like nuclei (arrowheads) observed in the ΔMcph1 cell lines. The nuclei were visualized through DAPI staining. Scale bar: 10 μm. e, Quantification of the percentage of prophase-like nuclei cells. Data represent the mean of two independent experiments ± SD. A minimum of 134 cells was examined in each experiment. Two-sided Student’s t test. f, Cell-cycle analysis through propidium iodide flow cytometry in parental Mcph1+/+ and ΔMcph1 cell lines. g, Representative images from a normal-sized metaphase and a metaphase with hypercondensed chromosomes in ΔMcph1 cell lines. h, Mean length of all chromosomes in parental Mcph1+/+ and ΔMcph1 cell lines. The lengths were measured in arbitrary units (au); 15 metaphases were examined for each sample. One-way ANOVA followed by Dunnett’s test.

Following the protocol described by (Houlard et al., 2021), we generated mESCs with a deletion of exon 2 of the Mcph1 gene, consequently producing a gene knockout. Based on PCR genotyping, we selected 3 clones (#23_1, #70, #85) and confirmed the presence of the targeted deletion of exon 2 in clones #70 and #85 (Fig. 2a, b) by Sanger sequencing. We were unable to obtain satisfactory Sanger sequence data for clone #23_1 because of difficulties in resolving overlapping sequencing signal peaks of heterozygous deletions. However, this clone was included in further analysis. The absence of Mcph1 was confirmed by Western blotting for all subjected clones (Fig. 2c). For CRISPR/Cas9 off-targets analysis, we utilized NGS data from three Mcph1-knockout cell lines obtained previously by our group (unpublished data). We found no detectable off-target editing at the predicted sites (Supplementary Material 2).

It is known that dysfunction of Mcph1 is associated with an increased fraction of cells with prophase-like condensed (PLCs) chromosomes in interphase (Arroyo et al., 2017; Houlard et al., 2021) (Fig. 2d). For the mutant clones we calculated the proportion of PLCs, which amounted to over 20–30 %, significantly differing from that in the parental Mcph1+/+ cell line (4 %) (Fig. 2e). Interestingly, this significant disruption of proper temporal activation of chromosome condensation does not affect the cell cycle progression. The proportion of cells in different stages of the cell cycle was similar in both parental Mcph1+/+ and ΔMcph1 cell lines, which is consistent with previous findings (Arroyo et al., 2017; Houlard et al., 2021) (Fig. 2f ).

Additionally we measured the metaphase chromosome length from ΔMcph1 cell lines and compared it to the parental line. Our analyses demonstrate that metaphase chromosomes in Mcph1-depleted cells are significantly shorter than the chromosomes of the parental line (Fig. 2g, h).

Moreover, we observed a significant increase in micronuclei in Mcph1-lacking cells (Supplementary Material 3). In two out of three ΔMcph1 cell lines we detected the formation of a Robertsonian metacentric chromosome by the fusion of two acrocentric chromosomes (marked by the red arrowhead at Fig. 2g).

Effects of Mcph1 knockout on gene expression in mESCs

**Fig. 3.**
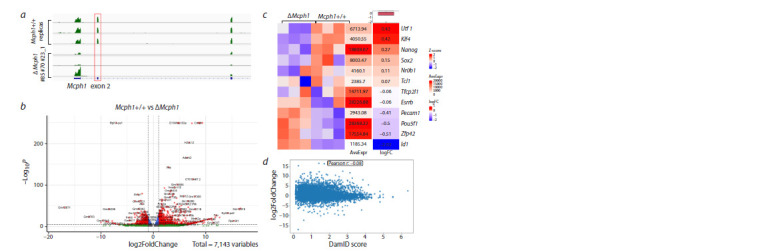
Effects of Mcph1 depletion on gene expression in mESCs. a, RNA sequencing coverage across the first three exons of Mcph1 in ΔMcph1 cell lines. b, Volcano plot of the significant DEGs between parental cell lines and ΔMcph1 cell lines. The x-axis represents the log2 fold change and the y-axis represents –log10 of each significant DEG. Red spots beyond the dashed lines are considered to be significantly expressed at p ≤ 0.05. c, Heat map of pluripotency gene expression values for all the cell lines used. Each horizontal line represents a gene and each column represents a single sample. The color intensity reflects the level of gene expression (red for upregulation and blue for downregulation). d, The correlation between DEGs and LADs. For the gene sets with significantly altered gene expression after Mcph1 knockout (log2 fold change y-axis) DamID contact frequency scores are shown (x-axis).

To determine if specific interphase chromatin features affect gene expression, we conducted a transcriptome analysis in the ΔMcph1 cell lines and the control parental Mcph1+/+ cell line. RNA-seq also confirmed the deletion of exon 2 of the Mcph1 gene in all targeted cell lines (Fig. 3a). The absence of transcripts aligning to the second exon in ΔMcph1 cell lines unequivocally indicates successful CRISPR/Cas9-mediated targeting. According to RNA-seq data, the expression level of Mcph1 in knockout cell lines decreases threefold ( p- value = 1.05e–15) compared to the parental cell line, likely due to the activation of the nonsense-mediated RNA decay mechanism (Brogna, Wen, 2009).

To determine the changes in gene expression following Mcph1 knockout, we analyzed RNA-seq data from three independently derived knockout cell lines and compared them with three replicates of the parental cell line. Genes with a base mean expression <100 were excluded from analysis. We found that 876 genes significantly changed their expression level (twofold or more) after Mcph1 knockout (see Supplementary Material 1 for the whole list of differentially expressed genes (DEGs)). These DEGs are equally distributed between up- and downregulated genes’ groups. Classification by Gene Ontology (GO) terms revealed 5 significantly-affected categories (FDR p-value < 0.05) related to general metabolism and olfactory receptor activity (see the Table). Interestingly, terms of sensory perception were not attributed to Mcph1 knockout before. While oxidative phosphorylation was highlighted as one of the most affected pathways in primary cultures of neural progenitors from Mcph1 full-knockout mice (Journiac et al., 2020).

**Table 1.**
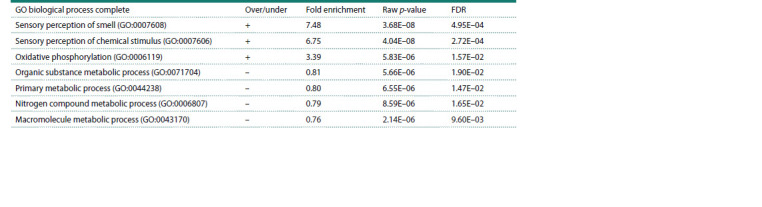
Gene Ontology categories with FDR <0.05 enriched after Mcph1 knockout in mESCs

We did not observe an enrichment of regulated genes associated with cell cycle control – pathways in which Mcph1 is known to be involved (Yang et al., 2008). In detail, there were no significant differences in the expression levels of Chk1, Brca1, Topbp1, Ddb2, p73 and Tert, which were all reported to show reduced expression following Mcph1 knockout (Yang et al., 2008). Contrary to previous reports, we observed a slight but significant upregulation of Rad51 and Apaf1 expression level in ΔMcph1 cell lines (log2FoldChange = –0.81, adjusted p-value =3.27×10–9 for Rad51; log2FoldChange = –0.6, adjusted p-value = 6.38×10–5 for Apaf1).

One of the hallmarks of embryonic stem cells is their ability to differentiate into almost any cell type. Thus, the high number of differentially expressed genes between parental Mcph1+/+ and ΔMcph1 cell lines might be a consequence of cell differentiation after Mcph1 depletion. To test this hypothesis, we have further analyzed the expression levels of key pluripotency markers such as Sox2, Pou5f1, Nanog, Klf4, etc. We have not observed any significant or consistent decrease in expression of these genes thereby indicating that differentiation had not taken place (Fig. 3с). Thus, Mcph1 is involved in the regulation of pluripotency in mESCs neither directly nor indirectly through influencing global chromatin organization.

The Mcph1 depletion induces significant remodeling of nuclear chromatin due to chromosome condensation. It can be hypothesized that the formation of rod-shaped chromosomes during interphase may cause disruptions in chromatin association with the nuclear lamina. Thus, we decided to find a correlation between alterations in gene expression level and frequency of contact with the lamina. Lamina-associated domains (LADs) regions in mESCs were identified by DamIDseq of Lamin B1 (Borsos et al., 2019). We found no correlation between the DamID contact frequency scores and changes in gene expression in mutant Mcph1 cells (Fig. 3d). These data suggest that Mcph1-mediated premature chromosome condensation during interphase is not the one that leads to changes in gene expression patterns of mESCs.

## Discussion

Microcephalin (Mcph1) is found in all metazoa. This multifaceted protein plays an important role in multiple fundamental cellular processes including DNA damage repair, cell-cycle progression and apoptosis, regulation of chromosome condensation and centrosome biogenesis. Loss-of-function mutations of Mcph1 cause primary microcephaly, associated with severe reduction in brain volume and clinical decline in neurocognitive function (Jackson et al., 2002). Previous studies have shown that the expression level of Mcph1 is decreased in many types of cancers including breast cancer, lung cancer, cervical cancer, etc. compared to normal tissue (Alsolami et al., 2023). Thus, Mcph1 has attracted intense research interest due to its crucial role in neurogenesis and cancer suppression (Pulvers et al., 2015; Liu et al., 2016).

Numerous studies have implied that Mcph1 plays an important role in chromosome maintenance (Arroyo et al., 2017; Cicconi et al., 2020). Tracking the dynamics of mitosis progression in Mcph1-depleted cells in real time revealed a range of anaphase defects and missegregated chromosomes that become encapsulated in micronuclei (Arroyo et al., 2017). Mcph1 specifically interacts with TRF2 in the shelterin complex of telomeric DNA and promotes homology-directed repair of dysfunctional telomeres. Moreover, Mcph1 supports telomere replication during the S-phase of the cell cycle by counteracting replication stress (Cicconi et al., 2020). In our study we also observed an elevated frequency of chromosomal abnormalities including micronuclei and Robertsonian translocations in the knockout ΔMcph1 lines (Supplementary Material 3). According to the previously published data we also detected a significant reduction in chromosome length for all ΔMcph1 cell lines (Gruber et al., 2011; Arroyo et al., 2017) (Fig. 2h). A similar phenomenon of hypercondensed metaphase chromosomes was also observed in cells continuously treated with nocodazole resulting in spindle destruction and significant prolonged mitosis (Naumova et al., 2013). Thus, increasing the duration of condensin loading to chromatin either by prolonged metaphase arrest after nocodazole treatment or chromosome condensation in interphase nuclei mediated by Mcph1 knockout leads to the shortening of mitotic chromosomes.

Several studies reported the transcriptional activity of MCPH1 (Lin, Elledge, 2003; Yang et al., 2008; Shi et al., 2012). It was shown that in HEK293 cells MCPH1 acts as a coactivator by forming a complex with the transcription factor E2F1 and regulates a number of genes (such as CHK1 and BRCA1) involved in DNA repair, the cell cycle and apoptosis (Yang et al., 2008). Furthermore, MCPH1 was first identified as an inhibitor of hTERT expression – that is why MCPH1 is also called BRIT1 (BRCT-repeat inhibitor of TERT expression) (Lin, Elledge, 2003). Later it was demonstrated that MCPH1 directly binds to the hTERT proximal promoter leading to reduced hTERT expression and telomerase activity (Shi et al., 2012). Comparative gene expression profiling of neural progenitors in Mcph1 knockout and wild-type mice has revealed altered expression of genes controlling the cell cycle and genes related to metabolic pathways (Journiac et al., 2020). In our study we investigated the changes in the transcriptional profiles of mESCs after Mcph1 knockout. Among significantly upregulated and downregulated (876) DEGs, GO analysis revealed enrichment for general metabolism and sensory perception of smell. Although it is hard to draw direct connections to the known Mcph1 functions, these data show that mESCs may try to adapt their metabolism to chronic chromatin hypercondensation. Furthermore, contrary to the aforementioned studies, we found no significant differences in the expression levels for Tert or for genes implicated in the cell cycle pathway after Mcph1 knockout in mESCs. Thus, contribution of Mcph1 to the regulation of gene expression appears to be species- and tissue-specific. This is also supported by the fact that most of the human-specific amino acid substitutions in MCPH1 resulted in changes in the regulatory effects on the downstream genes (Shi et al., 2013).

It is now established that spatial organization of chromatin in the nucleus is important for proper regulation of gene expression. Mcph1 knockout results in the loading of condensin II onto chromatin followed by chromosome condensation during interphase. It is possible to assume that at least a part of the expression changes after Mcph1 knockout could be explained by alterations in chromatin spatial organization. It was previously shown that condensin II depletion contributes to the folding of the human genome by shifting from chromosome territories to Rabl-like polarized organization with chromocenter formation (Hoencamp et al., 2021). Such drastic reorganization affects the expression of a small fraction of genes within LADs and near LAD borders (Hoencamp et al., 2021). Knockout of Mcph1 also leads to large-scale reorganization but in the opposite manner: interphase chromosomes are individualized into prophase-like rod-shaped chromatids, while chromocenters have disappeared. In our transcriptome analysis of mESCs with Mcph1 knockout, we found no correlation between changes in the expression level of genes and their proximity to lamina. Thus, loading of condensin II onto chromatin does not affects smaller-scale chromatin structures such as LADs and TADs (topology associated domains) contributing to the regulation of gene expression.

## Conclusion

In this work we have generated mESCs with a knockout of the Mcph1 gene. Our conclusion is that Mcph1 is likely not involved in the regulation of gene expression in mESCs by direct binding to target promoters or by modulation of spatial chromatin organization, while the DEGs observed may be the result of secondary effects due to persistent chromatin hypercondensation. These cell lines will be a valuable resource for investigating Mcph1-condensin II pathway in chromosome maintenance, and could also be used to study Mcph1 roles in DNA repair.

## Conflict of interest

The authors declare no conflict of interest.
